# Quantifying surface morphology of manufactured activated carbon and the waste coffee grounds using the Getis-Ord-Gi* statistic and Ripley’s K function

**DOI:** 10.1038/s41598-021-87249-0

**Published:** 2021-04-06

**Authors:** Sanghoon Lee, Sukjoon Na, Olivia G. Rogers, Sungmin Youn

**Affiliations:** 1 Department of Computer Sciences and Electrical Engineering, Marshall University, Huntington, 25755 WV USA; 2 Department of Civil Engineering, Marshall University, Huntington, 25755 WV USA; 3 Department of Mechanical Engineering, Marshall University, Huntington, 25755 USA

**Keywords:** Engineering, Materials science, Mathematics and computing

## Abstract

Activated carbon can be manufactured from waste coffee grounds via physical and/or chemical activation processes. However, challenges remain to quantify the differences in surface morphology between manufactured activated carbon granules and the waste coffee grounds. This paper presents a novel quantitative method to determine the quality of the physical and chemical activation processes performed in the presence of intensity inhomogeneity and identify surface characteristics of manufactured activated carbon granules and the waste coffee grounds. The spatial density was calculated by the Getis-Ord-Gi* statistic in scanning electron microscopy images. The spatial characteristics were determined by analyzing Ripley’s K function and complete spatial randomness. Results show that the method introduced in this paper is capable of distinguishing between manufactured activated carbon granules and the waste coffee grounds, in terms of surface morphology.

## Introduction

Activated carbon is one of the most widely used absorbents in water and wastewater treatment^1^. Activated carbon has been used since ancient Greece to remove contaminants in air and water^2^. Relatively small amounts of activated carbon can absorb significant amounts of contaminants due to its extremely large surface area per unit mass. The porous structure of activated carbon determines the absorptive capacity^3^. Many studies have shown the effectiveness of activated carbon adsorption for a wide range of contaminants. According to the literature, activated carbon can effectively adsorb both organic and inorganic contaminants including biological nutrients, pharmaceuticals, endocrine disruptors, pesticides, dyes, heavy metals, and even persistent organic pollutants like per- and polyfluoroalkyl substances^4–11^. In recent years, activated carbon has also received considerable attention as a reinforcement of cement-based composites such as a concrete mixture. Mahoutian et al.^12^ reported that activated carbon reduced the air content in concrete, resulting in an increase of strength. Chowdhury et al.^13^ found that activated carbon promoted moisture resistance of cement and mitigated the risk due to moisture attack.

Although activated carbon is advantageous in diverse engineering applications, the use of activated carbon can be expensive as spent activated carbon has to be replaced with new carbon over time. Wood, coal, and lignite are the most common base materials for activated carbon production and less than 20% of the total production utilizes renewable base materials^14,15^. The literature suggests that activated carbon can be prepared from alternative biomass including agricultural by-products, manure, wastewater sludge that are not coal-based resources^14,16–18^. Challenges, however, still remain to utilize activated carbon obtained from alternative biomass sources. For example, the quality of activated carbon manufactured from biomass is highly dependent on the activation process, and inappropriate processing may lead to inferior surface characteristics of activated carbon. To ensure and produce activated carbon that meets the specification, a method that can quantify the surface morphology of activated carbon is demanded because the adsorption capacity of activated carbon is strongly correlated with the surface characteristics such as the pore irregularity and the surface roughness. Fractal analysis that analyzes fractal dimensions of the surface of activated carbon has been widely used by many studies^19–23^. However, the application of fractal analysis often produces biased or incorrect results due to insufficient data points or errors in regression analysis^24^. Furthermore, the fractal dimension analysis may give the same fractal number to two different objects and, thus the fractal dimension of a set of objects is not sufficient to describe the difference between each object^25^. In addition to fractal dimension, specific surface areas of fractal surfaces are experimentally estimated by the Brunauer–Emmett–Teller (BET) theory for activated carbon^26^. However, BET measurements can be also unreliable for some carbonaceous materials, especially for biochars, as degassing process alters the material properties^27^.

In this paper, we present a seamless solution that quantitatively measures the quality of activated carbon manufactured by the physical and chemical activation process and identifies surface characteristics of both activated carbon granules and the waste coffee grounds through scanning electron microscopy (SEM) images. The Getis-Ord-Gi* statistic is used to identify patterns of the spatial significance in the SEM images. Six feature detection algorithms, Minimum Eigenvalue (ME)^28^, Harris-Stephens (Harris)^29^, Binary Robust Invariant Scalable Keypoints (BRISK)^30^, Speed up Robust Feature (SURF)^31^, KAZE^32^, and Oriented fast and Rotated Binary (ORB) robust independent elementary features^33^, were analyzed to determine point-patterns in a two-dimensional space. Differences in surface morphology between manufactured activated carbon granules and the waste coffee grounds were measured by quantifying the area of a region between Ripley’s K function and complete spatial randomness (CSR).

## Result and discussion

**Figure 1:**
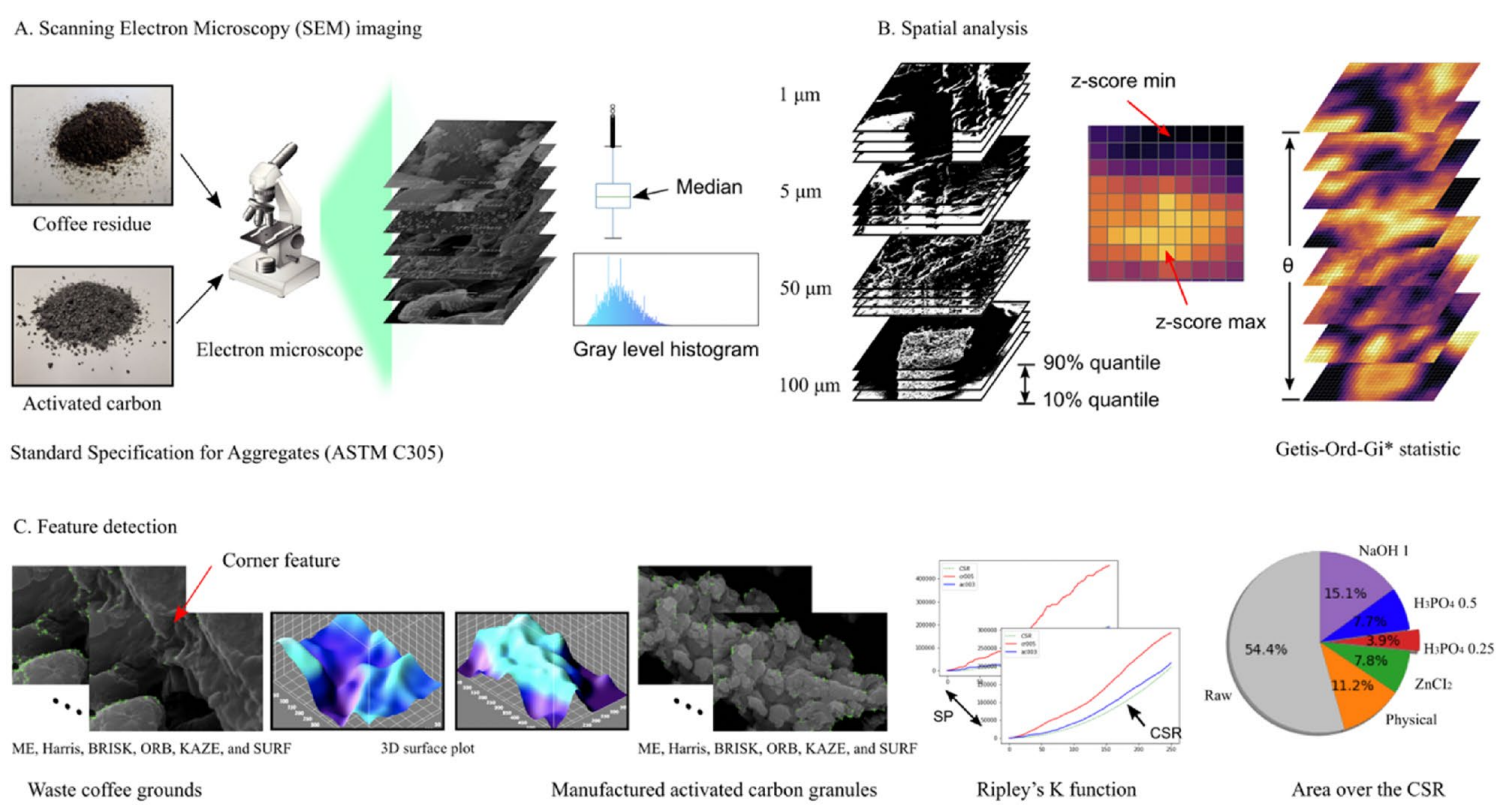
A quantitative process of manufactured activated carbon granules and the waste coffee grounds. (**A**) Manufactured activated carbon granules and the waste coffee grounds were captured by a scanning electron microscope (**B**) A spatial analysis was performed to identify the spatial characteristics of the point-patterns over the SEM images. (**C**) Features of the SEM images were detected by six feature detection algorithms. Differences in surface morphology were measured by quantifying the area of a region between Ripley’s K function and CSR.

A quantitative process of manufactured activated carbon granules and waste coffee grounds using the Getis-Ord-Gi* statistic and Ripley’s K function is described in Fig. 1. The magnifications of the SEM images are 75×, 500×, 5000×, and 20,000×. To determine spatial characteristics of the point-patterns in the SEM images, the Getis-Ord-Gi* statistic was calculated (Fig. 1B). Features in the SEM images (600 × 400 pixels) were detected by six feature detection methods: ME, Harris, BRISK, ORB, SURF, and KAZE. Ripley’s K function was employed to analyze the detected features(Fig. 1C).

**Figure 2:**
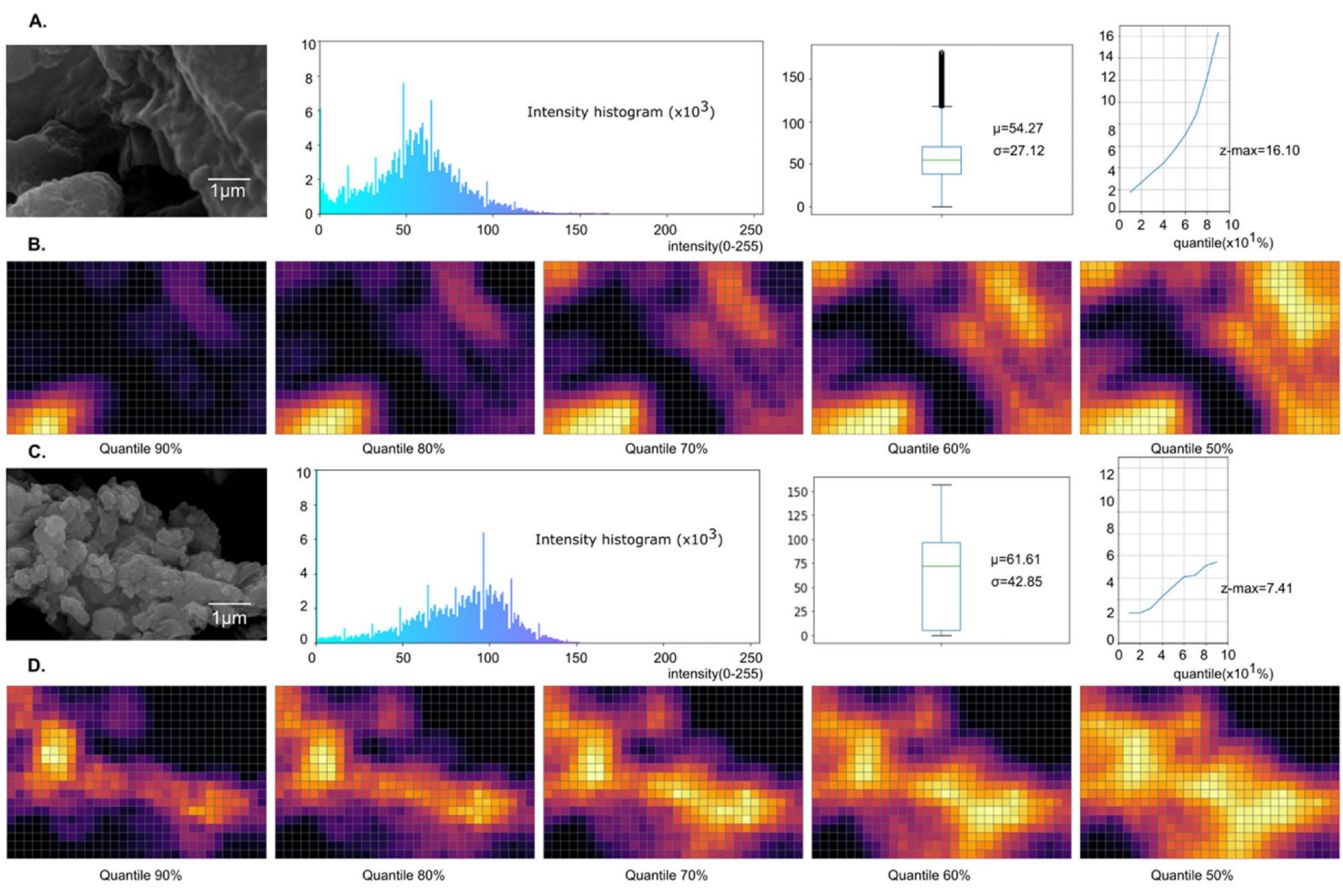
Getis-Ord-Gi* statistic of the SEM images. (**A**) (Left to right) A SEM image captured from the waste coffee grounds. Intensity histograms of the image. A box plot of the histogram. Z-score plots with the quantiles from 10 to 90%. We validated differences in quantiles using z-scores. (**B**) Heatmaps with 50%, 60%, 70%, 80%, 90% quantiles. (**C**) (Left to right) A SEM image captured from manufactured activated carbon granules. Intensity histograms of the image. A box plot of the histogram. Z-score plots with the quantiles from 10 to 90%. (**D**) Heatmaps with 50%, 60%, 70%, 80%, 90% quantiles.

Eight SEM images for each activated carbon and waste coffee ground sample were captured at four different magnification levels. The Getis-Ord-Gi* statistic was then used to generalize the pixel-wide regions beyond the uniform maps and represent the density of the regions (Fig. 2). Figure 2A represents a raw SEM image for the waste coffee grounds at 20,000×, an intensity histogram of the image, a box plot of the intensity values, and a z-score graph with different quantiles (Supplement Table S1 and S2). Figure 2B,D illustrate two-dimensional hotspot regions generated by the Getis-Ord-Gi* statistic with varying ratios of the quantile. Figure 2C represents a raw SEM image for manufactured activated carbon granules with 20,000×. The analysis of the Getis-Ord-Gi* statistic indicates that (1) the maximum z-scores of the waste coffee grounds is greater than those of the manufactured activated carbon granules. (2) the z-scores of waste coffee grounds tend to be steeper slopes compared to those of activated carbon as it increases the quantile percentages. Therefore, the density level can be a factor distinguishing between the surface of manufactured activated carbon granules and the waste coffee grounds by using Getis-Ord-Gi*.

Although the Getis-Ord-Gi* statistic can be used for the analysis of the surface morphology, it is only applicable for limited regions. For example, raw SEM images may contain arbitrary granular materials with the background regions. These regions often prevent the constant dispersion measure, which is essential for the potential identification and characterization of the surface of granular materials. To overcome these limitations, feature detection methods were used to identify features from the overall region.

Features identified by the feature detection methods are shown in Supplement Fig. 1. Each column represents the feature detection method and each row represents the SEM images of manufactured activated carbon granules (A1, A2, A3, and A4) and the waste coffee grounds (B1, B2, B3, and B4) based on the magnifications: 20,000×, 5000×, 500×, and 75× (top to bottom). We found that the features marked with green color at the second row and the fourth column are more well-distributed than the features marked with green color at the sixth row and the fourth column. This result indicates that (1) the features identified by BRISK are distinguishable factors between manufactured activated carbon granules and the waste coffee grounds and (2) there was a difference between the feature detection methods. However, the comparison relying on intuition only potentially causes an inter-observer variation influenced by systematic bias on observer experiences. To avoid the problem of intuition, we created the 95% confidence interval error ellipse to specify the error variance for the detected features. The covariance matrices to represent the variability of the feature points in the surface of the manufactured activated carbon granules and the waste coffee grounds were measured accordingly and described in Supplement Table S3.

**Figure 3:**
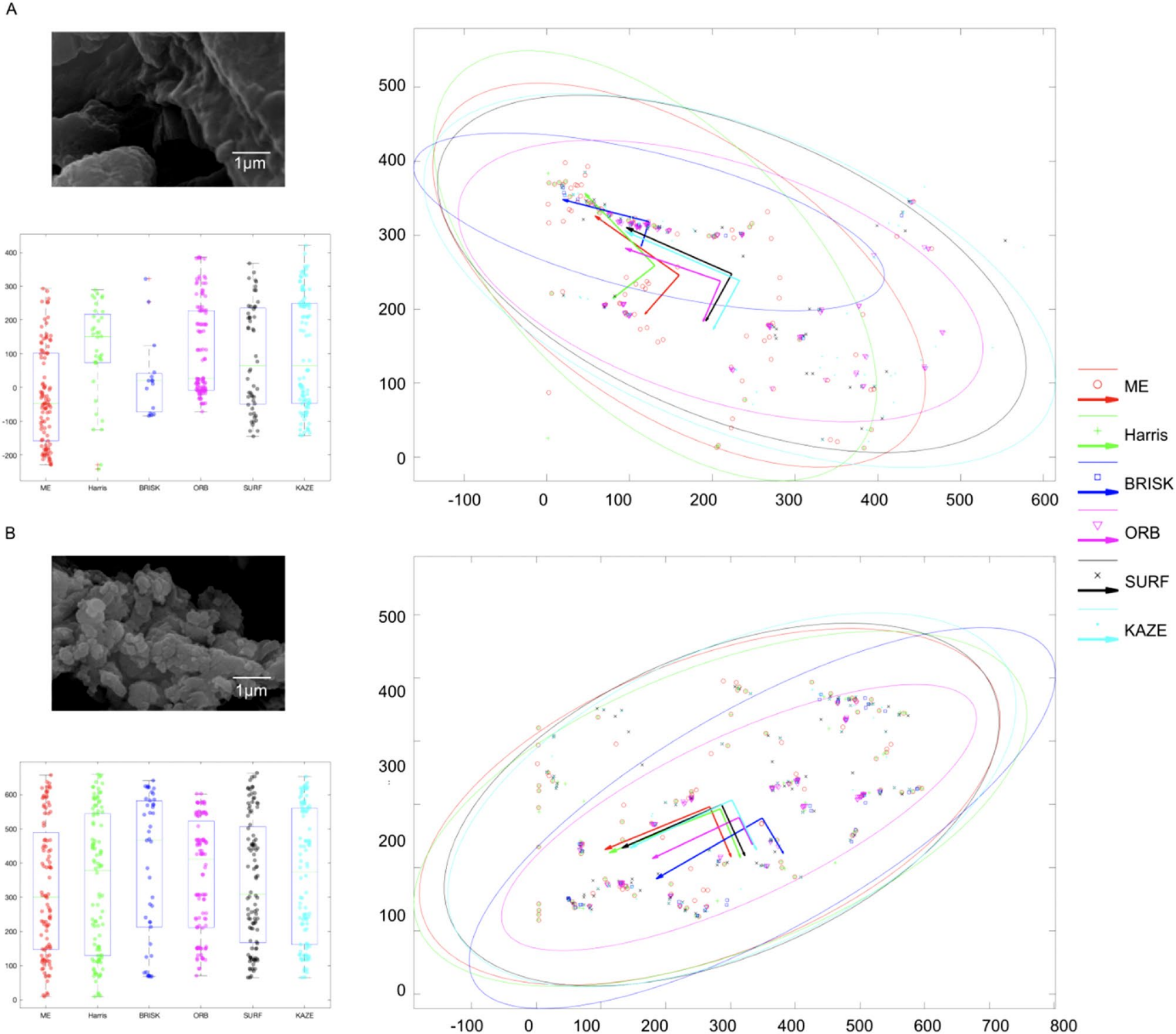
Confidence interval error ellipses and feature distributions of the SEM images. (A) An SEM image was captured from the waste coffee grounds (top left). The distribution of features detected by six feature detection algorithms. The two-dimensional space of the covariance matrix of feature points was reduced to one-dimensional space using Principal Component Analysis (PCA) (bottom left). The 95% confidence interval error ellipse for the detected features. A list of markers was used to represent the features detected by the algorithms (ME: red circle, Harris: green plus sign, BRISK: blue square, ORB: pink downward-pointing triangle, SURF: black cross, and KAZE: light blue dot. The features inside the ellipse represent the ones that contain 95% of all features based on the Gaussian distribution (right). (B) An SEM image was captured from manufactured activated carbon granules (top left). The distribution of features detected by six feature detection algorithms (bottom left). The 95% confidence interval error ellipse for the detected features (right).

The 95% confidence interval error ellipses and feature distributions of the SEM images are shown in Fig. 3. We used the identical SEM images from both manufactured activated carbon granules and the waste coffee grounds. The results of the 95% confidence interval error ellipses show that the eigenvectors of the covariance matrix of the features identified from B are more likely in the same direction than the ones of the features identified from A for two-dimensional normally distributed features. Moreover, we performed the dimensionality reduction on the two-dimensional feature data by reducing the number of dimensions from two to one using principal component analysis (PCA). The results indicate that the one-dimensional features identified from manufactured activated carbon granules are evenly distributed, but the one-dimensional features identified from the waste coffee grounds are unevenly distributed.

**Figure 4:**
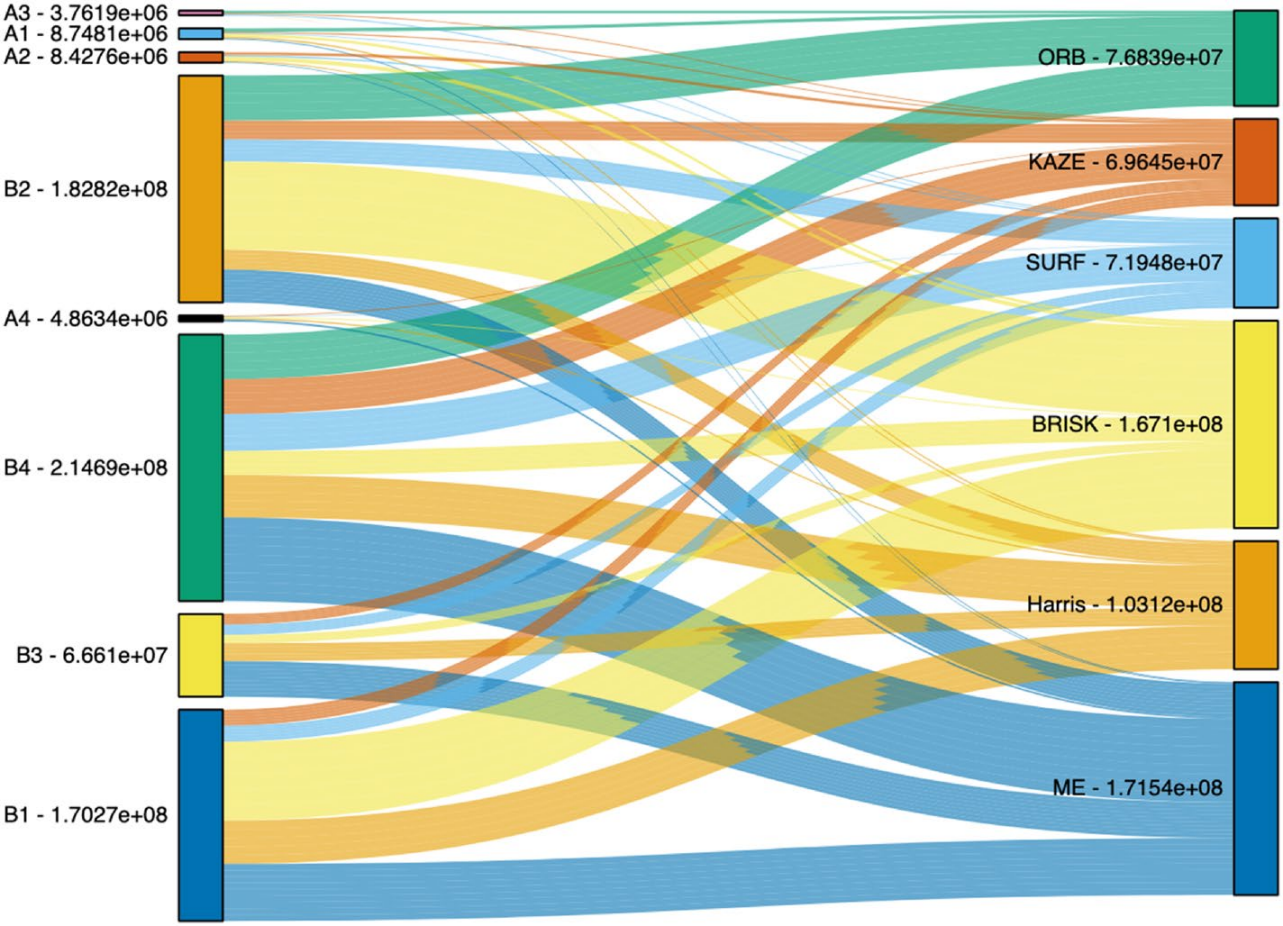
Quantitative measuring and influencing mechanism of Area of a region between Ripley’s K function and CSR**.** (Left) SEM images captured from manufactured activated carbon granules and waste coffee grounds. A1, A2, A3, and A4 are SEM images captured from manufactured activated carbon granules with different resolutions, while B1, B2, B3, and B4 are SEM images captured from waste coffee grounds with different resolutions. The sum of the areas between the graphs of the functions represents the height of the left box. (Right) Feature detection algorithms: ME, Harris, BRISK, KAZE, SURF, and ORB. The sum of the areas between the graphs of the functions represents the height of the right box. The width of the band is proportional to the area of a region between Ripley’s K function and CSR.

Characteristics of the SEM images can be identified by capturing the essence of an image pattern. Each SEM image prepared from the previous approaches was analyzed either to obtain a list of features by the feature detection methods or to compute the statistics of the 20 × 20 grid region. In addition to these approaches, we investigated another approach to find the differences in surface morphology between manufactured activated carbon granules and the waste coffee grounds. The main contribution of our paper is that we quantified the impact of the features identified by the feature detection methods by measuring the area of a region between Ripley’s K function and CSR often called a homogeneous spatial Poisson process. A statistical test against the homogeneous spatial Poisson process was performed by estimating the variance of the features by using Ripley’s K function. Ripley’s K function computes features’ concentration based on a range of distance or scales so that the relationships between the features can be fully identified. The results of the quantitative measuring and influencing mechanism of the area of a region between Ripley’s K function and CSR are shown in Fig. 4. A1, A2, A3, and A4 are the SEM images captured from manufactured activated carbon granules, while B1, B2, B3, and B4 are SEM images captured from waste coffee grounds with different resolutions. The left side of the diagram in Fig. 4 represents input SEM images and the right side of the diagram represents the feature detection algorithms. The width of the band represents the area of a region between Ripley’s K function and CSR. We quantified the difference between A2 and B2 by measuring the width of the bands in the BRISK. The area of a region between Ripley’s K function and CSR was measured to evaluate Ripley’s K functions of features identified by the feature detection methods. The sum of the areas between the graphs of the functions for each input SEM image corresponding to each feature detection algorithm is shown on the left side of the box. The flow within the diagram indicates that the SEM images captured from manufactured activated carbon granules cause small area, comparing with the SEM images captured from waste coffee grounds, indicating that the physical activation process is achieved by differentiating the quantity of the surface morphology with respect to manufactured activated carbon granules.

**Figure 5:**
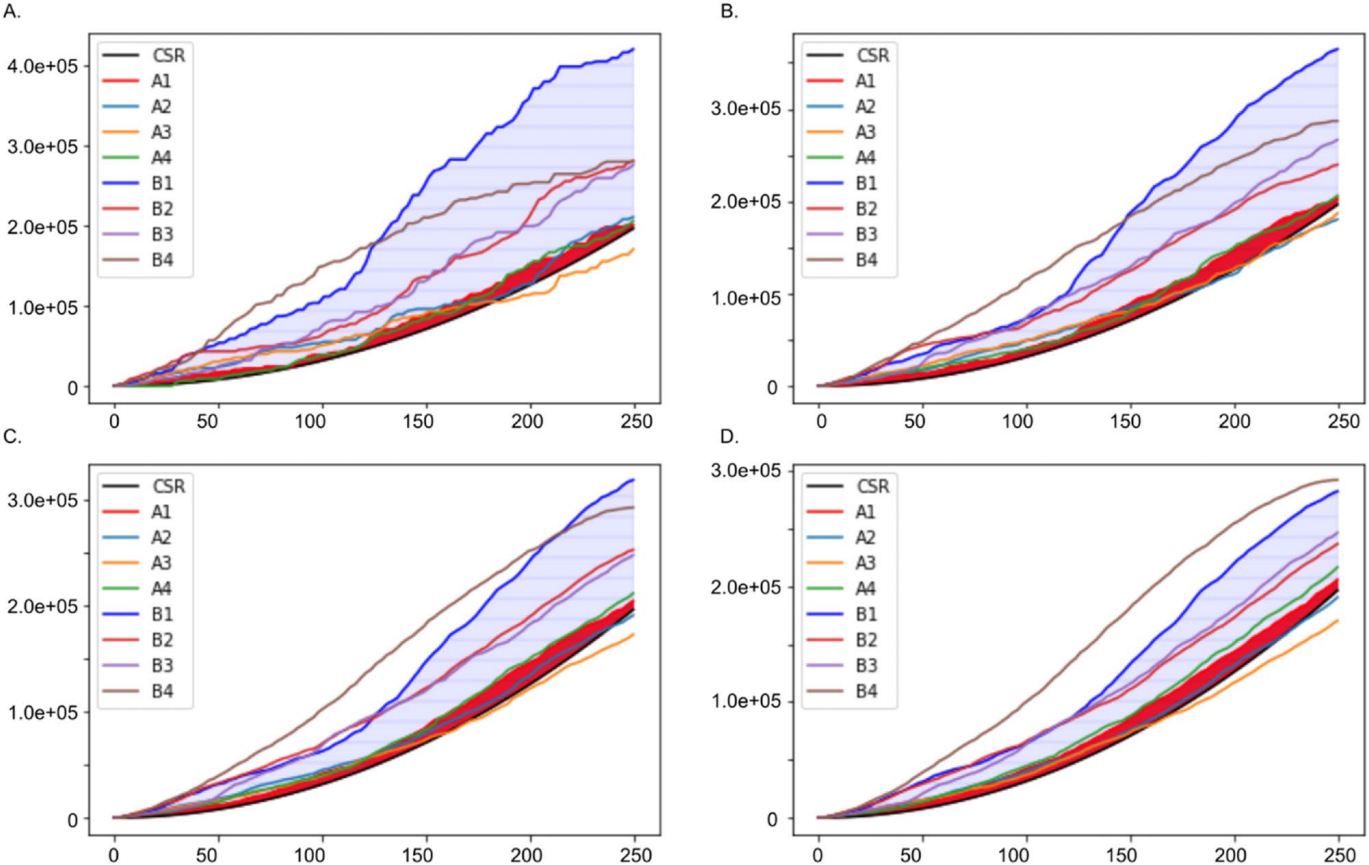
Ripley’s K functions and Complete Spatial Randomness(CSR) with different Strong Points of features (SPs). (**A**–**D**) Ripley’s K functions with 20, 40, 60, and 80 SPs. The y-axis represents the K-function value and the x-axis represents the radius of point-patterns. The lighter blue shaded area represents the area of a region between Ripley’s K function and CSR for B1, while the red shaded area represents the area of a region between Ripley’s K function and CSR for A1.

The presented quantitative measurements in surface morphology between manufactured activated carbon granules and the waste coffee ground vary in the number of strong points of the features identified by the feature detection methods. Four experiments were performed on the presented approach with different strong points: 20, 40, 60, 80. The variance of the strong points was estimated by Ripley’s K function and compared with the CSR. In Fig. 5, the CSR plot was simulated in green-dash lines. Ripley’s K functions for two surface configurations of the spatial points (A1 and B1) were simulated in two lines: red-solid and blue-solid. The results show that (1) Ripley’s K function for the surface of manufactured activated carbon granules are more likely to follow the CSR, which indicates that the dispersion of manufactured activated carbon’s surface is substantially higher than the one from the waste coffee ground’s surface and (2) the higher SPs surfaces are more distinguishable than those in lower SPs.

**Figure 6:**
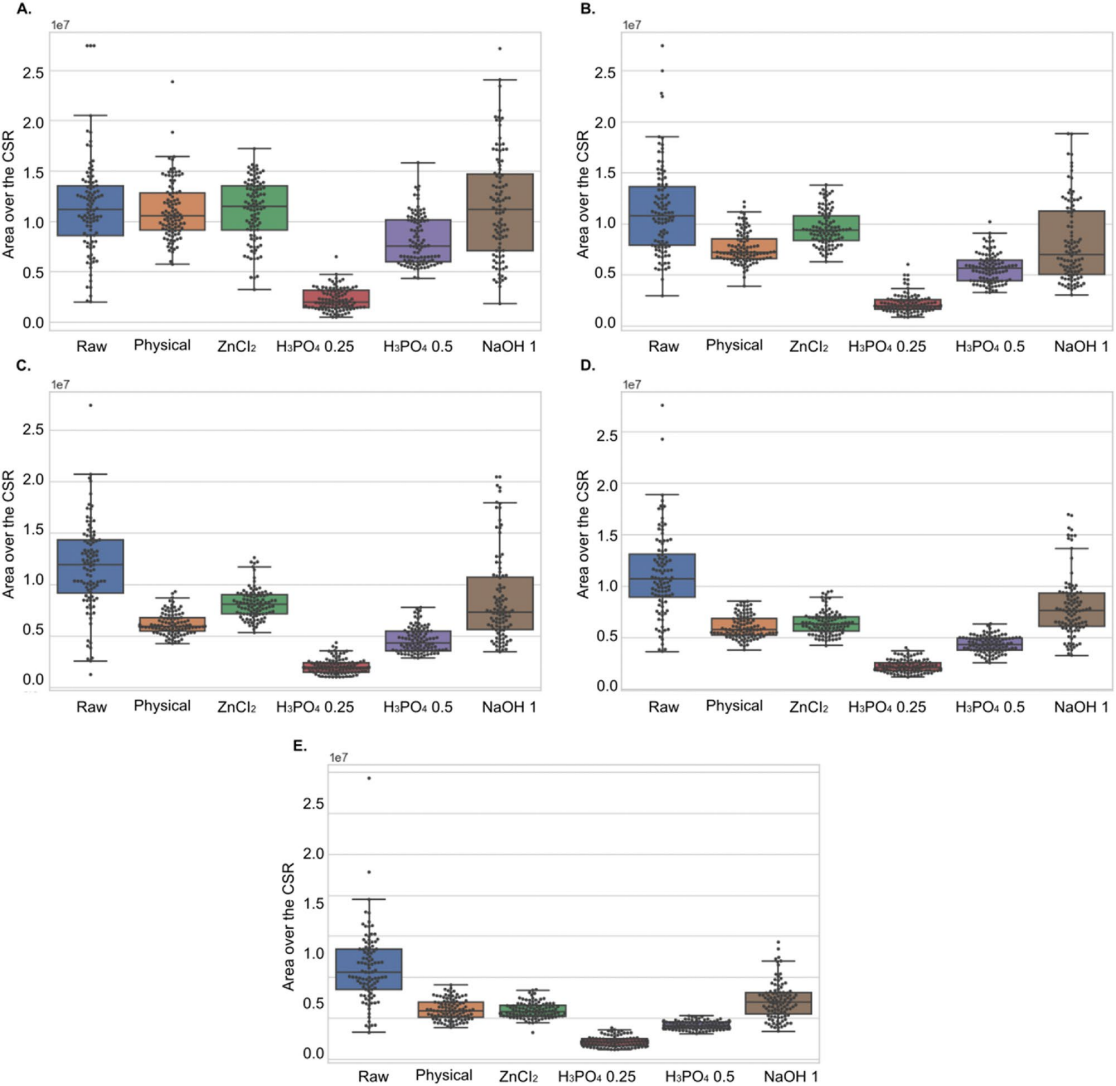
Quantitative measuring of the area of a region between Ripley’s K function and CSR on Strong Points of features (SPs). (**A**–**E**) Box plots for 20, 40, 60, 80, and 100 SPs. The y-axis represents the area over the CSR and the x-axis represents the augmented SEM images for the waste coffee grounds and the manufactured activated carbon granules.

We performed an additional experiment on the waste coffee ground (raw) and activated carbon granules manufactured via physical activation and chemical activation using three chemical agents (i.e., H_3_PO_4_, ZnCl_2_, and NaOH) shown in Supplement Fig. 2 and Supplement Table S4. However, a limited number of SEM images can be a major obstacle in verifying the quantitative process. We augmented the SEM images used in Supplement Fig. 2 by 360-degree random rotation, generating 600 SEM images with random rotation augmentation. The features of each SEM image were identified by 6 feature detection methods, and then the area of a region between Ripley’s K function and CSR for 5 SPs was computed. The quantitative results of the augmented SEM images are illustrated as box plots in Fig. 6. The box plots show that the area of a region between Ripley’s K function and CSR in H_3_PO_4_ are smaller and denser than those in Raw. All results are illustrated in Supplement Table S5. These results indicate that the presented approach is effective to distinguish the SEM images captured from the manufactured activated carbon granules from the SEM images captured from the waste coffee grounds.

In this paper, we presented a way of analyzing the surface morphology of manufactured activated carbon and the waste coffee grounds using the Getis-Ord-Gi* statistic and Ripley’s K function. Quantifying surface morphology in manufactured activated carbon granules has been a challenge in diverse engineering applications. We used Getis-Ord-Gi* statistic to identify spatial characteristics of the point-patterns in a 20 × 20 grid region over the SEM images with different magnification. However, raw SEM images with arbitrary granular materials often prevent the constant dispersion measure from the potential identification and characterization of the surface of granular materials. To avoid this issue, features extracted from the SEM images were also used to determine the spatial characteristics of manufactured activated carbon granules and waste coffee grounds. Ripley’s K function is adopted to evaluate the spatial characteristics of the features identified by the feature detection methods. These experiments provide clear evidence that the presented method is capable of distinguishing between manufactured activated carbon granules and the waste coffee grounds.

Although the presented method performs well on the high-quality SEM images with gray levels, our approach is limited to use both Getis-Ord-Gi* statistic and Ripley’s K function simultaneously because of arbitrary granular materials with the background regions, and it may have consequential functional limitations in either color images or three-dimensional images since the feature detection in a color image with three channels can be challenging to interpret the surface variability Fractal analysis can be adopted for the RGB images^34,35^ and three-dimensional images, but it often causes incorrect errors in regression analysis^24^. We plan to investigate any applicable method to overcome these limitations.

## Materials and methods

Waste coffee grounds were collected at a Starbucks coffee shop near Marshall University. Using 5-gallon plastic pails with lids, the spent coffee grounds were collected. The collected coffee waste consists of various types of wet coffee grounds. Activated carbon was produced from coffee waste in the laboratory following previously developed methods by other scholars^36–38^. The coffee grounds were first washed and rinsed with distilled water. The coffee waste was dried at 100 °C for 24 h in an oven and then cooled to room temperature in the laboratory. Each pail has a wide range of coffee grounds with various grain sizes, colors, and shapes. The dried coffee grounds are homogenized by gentle mixing. The dried coffee grounds were stored in a desiccator until the activation process to minimize possible adsorption of moisture. For the chemical activation process, three different chemical agents were used: H_3_PO_4_, ZnCl_2_, and NaOH. The dried coffee residue was first mixed in with either 1 M H_3_PO_4_, 1 M ZnCl_2_, or 1 M NaOH solution to various mass ratios (i.e., the mass of chemical agents to the mass of coffee grounds) in separate containers. The mixtures were given 24 h of contact time. The activation step was then followed where the mixtures were heated at 600 °C for an hour in a muffled furnace. For the physical activation, the prepared coffee grounds were directly heated at 600 °C for an hour without any chemical agent. After activation, activated carbon was washed, rinsed, and strained through U.S. standard No. 16 and 60 sieves to control grain-size (*i.e.*, larger than 250 µm and smaller than 1.18 mm). The American Society for Testing and Material (ASTM) classifies activated carbon particles having a size smaller than 0.180 mm as powdered activated carbon (PAC). Granular activated carbon (GAC) is defined as a minimum of 90% of the sample weight being retained on a 180-μm Standard sieve^39^. According to the ASTM definition, the lab-manufactured activated carbon is GAC.

The pore structures and surface morphology of activated carbon were examined using JEOL 5310LV and JEOL 7200FLV Scanning Electron Microscopes (SEM) at Marshall University. A small number of particles (approximately 10 particles) was applied to a piece of Carbon conductive double-sided tape. The other side of the tape was applied to a metal stub. The stub was securely affixed in a mount that was compatible with the SEM. All materials used, including tweezers and scissors, were cleaned before use with Ethanol (75% EtOH), and gloves were worn so as not to contaminate the sample or apparatus.

Minimum eigenvalue was used to determine affine changes based on a Newton–Raphson style minimization. We measured two small eigenvalues and two large eigenvalues representing the corners. Harris was utilized to measure the changes in intensity for the shift in an image patch such that distinctive image patches hold larger values while constant image patches hold smaller values. BRISK was used to compute non-maximal suppression across scale space. ORB was used to find strong points through the measurement of the Harris algorithm. We also detected features by using SURF finding the points over the intensity distribution of the pixels by shifting the neighborhood of the points of interest. All feature detection methods used in this paper were built-in functions available in MATLAB. Ripley’s K function is a point spatial analysis method that describes point patterns in the area of events. As the point pattern analyses have been utilized to elucidate the spatial arrangement of geographic points by clarifying relationships between point patterns, the estimation of Ripley’s K function is defined by:

$$\widehat{K}\left(r\right)=\frac{s}{{f}_{n}({f}_{n}-1)}\sum_{x}\sum_{y}I\left({d}_{xy}\le r\right){e}_{xy}$$

where $s$ is the size of the window, ${f}_{n}$ is the number of features, ${d}_{xy}$ is the distance between two features, $I\left({d}_{xy}\le r\right)$ is the indicator function that determines whether the distance ${d}_{xy}$ is less than or equal to $r$ which is a distance of a random selection, and ${e}_{xy}$ is Ripley’s isotropic edge correction weight. The area of a region between Ripley’s K function and CSR was approximated as the difference of the sum of all trapezoids. It is defined by:

$$Area\approx \sum_{i=1}^{n}[R\left({x}_{i}^{*}\right)-C({x}_{i}^{*})]\Delta x$$

where $R\left({x}_{i}^{*}\right)=(R\left({x}_{i-1}\right)+R\left({x}_{i}\right))/2$ and $C\left({x}_{i}^{*}\right)=(C\left({x}_{i-1}\right)+C\left({x}_{i}\right))/2. R\left({x}_{i}\right)$ is the height of Ripley’s function at ${x}_{i}$ and $C\left({x}_{i}\right)$ is the height of CSR at ${x}_{i}$. $\Delta x$ is the width if $i$-th subinterval.

## Supplementary Information

Supplementary Information 1.

Supplementary Information 2.

Supplementary Information 3.

Supplementary Information 4.

Supplementary Information 5.

Supplementary Information 6.

## Data Availability

All data are available from the corresponding author on request.
