# Supplementary material for: Quantifying surface morphology of manufactured activated carbon and the waste coffee grounds using the Getis-Ord-Gi* statistic and Ripley’s K function
Source: Sci Rep. 2021 Apr 6;11:7543. doi: 10.1038/s41598-021-87249-0 (PMC8024374; doi:10.1038/s41598-021-87249-0)
Supplement: Supplementary file 1 — Supplementary Information 1. [file 41598_2021_87249_MOESM1_ESM.pdf]

Quantifying surface morphology of manufactured activated carbon and the waste coffee grounds using the Getis-Ord-Gi* statistic and Ripley's K function

Sanghoon Lee[1], Sukjoon Na[2], Olivia G. Rogers[3], and Sungmin Youn[2]*

[1] Department of Computer Sciences and Electrical Engineering at Marshall University

[2] Department of Civil Engineering at Marshall University

[3] Department of Mechanical Engineering at Marshall University

*Corresponding Author: Weisberg Applied Engineering Complex Room 2207, One John Marshall Drive, Huntington, WV 25755; youns@marshall.edu; 1-304-696-6475.

SUPPLEMENTARY INFORMATION

SUPPLEMENTARY FIGURES

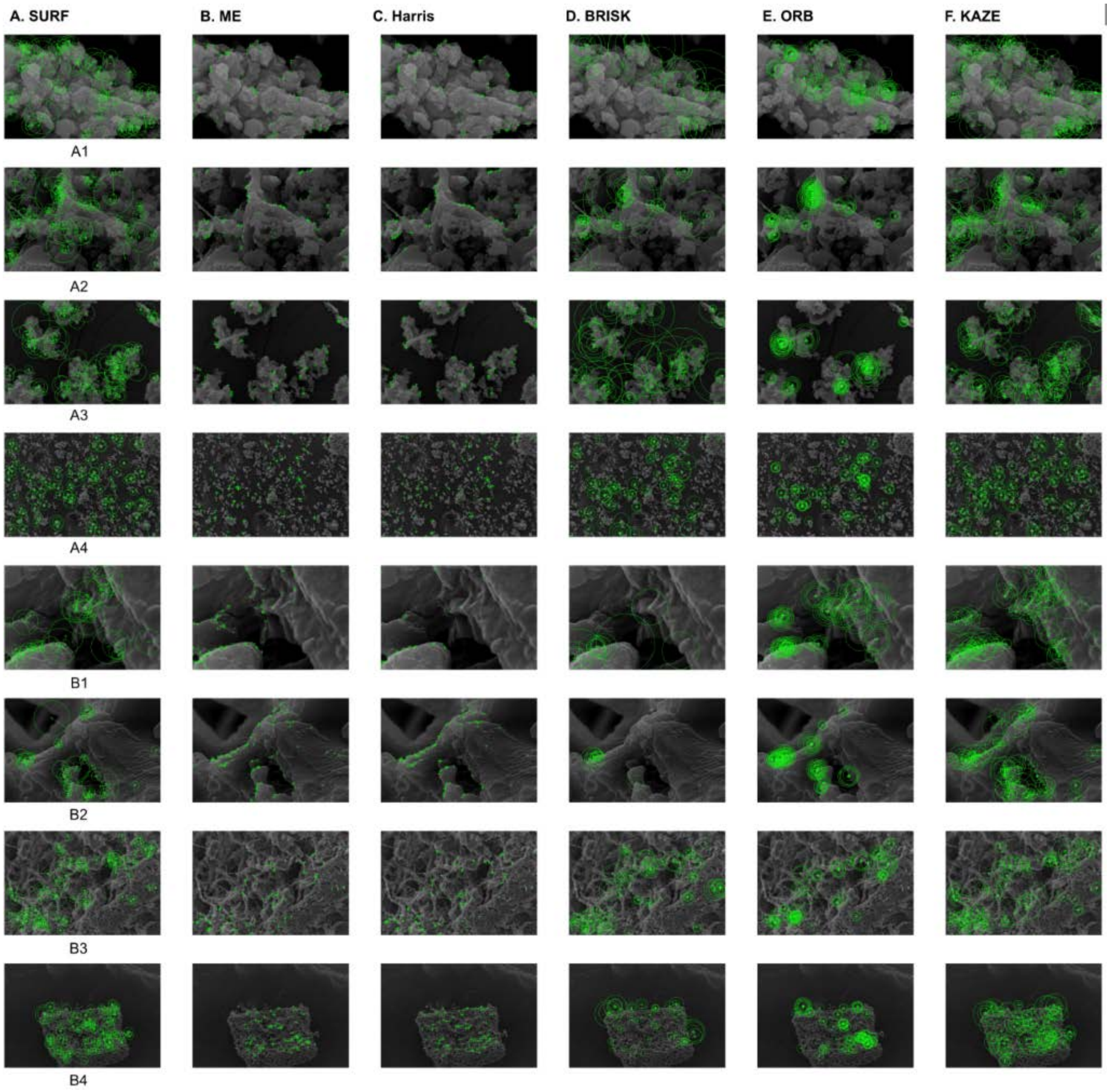

Supplement figure 1. The SEM images with the identified features using six feature detection algorithms. (Left to right) Feature detection algorithms: SURF, ME, Harris, BRISK, ORB, and KAZE. (Top to bottom) the SEM images captured from manufactured activated carbon granules and waste coffee grounds. A1, A2, A3, and A4 are SEM images captured from manufactured activated carbon granules with different resolutions, while B1, B2, B3, and B4 are SEM images captured from waste coffee grounds with different resolutions. The identified features were marked in green color referring to a distinct pattern from the surroundings in an image. Corner features were detected by ME, Harris, BRISK, and ORB. Blob features were detected by SURF and KAZE.

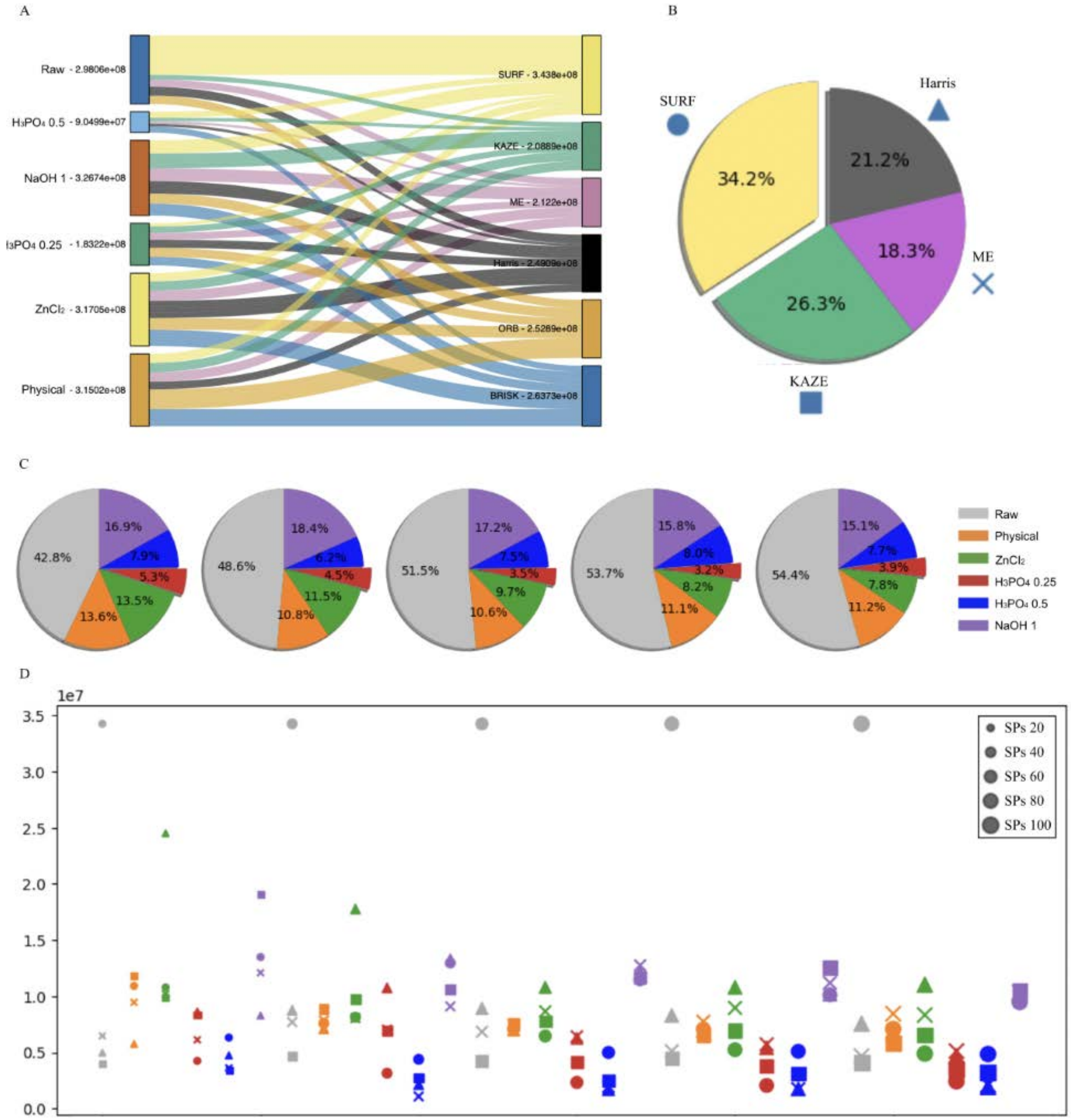

Supplement figure 2. Experiment results of quantitative measurement based on the area over the CSR. (A) Sankey diagrams represent the energy flow of the area of a region between Ripley's K function and CSR computed in the SEM images (1200 x 900 pixels) of Group A for six feature detection algorithms. (B) A pie box represents quantitative variables of the features detection algorithms in which the percentages are corresponding to the sum of the area over the CSR. Both BRISK and ORB were removed from the algorithms due to the 'NAN' values generated by Ripley's K functions. (C) Each pie box represents the percentages of the area over the CSR for the SEM images with 20 SPs, 40 SPs, 60 SPs, 80 SPs, 100 SPs (Left to right). (D) The areas over the CSR for the SEM images with different SPs were plotted. A list of makers (SURF: circle, KAZE: square, Harris: triangle, and ME: cross) was shown in the plot.

SUPPLEMENTARY DATASETS

Table S1. Intensity information of the SEM images: A1, A2, A3, A4, B1, B2, B3, B4.

Table S2. Getis-Ord-Gi* statistic: the maximum and the minimum z-scores with quantiles: 10%, 20%, 30%, 40%, 50%, 60%, 70%, 80%, 90%.

Table S3. Covariance matrix of the features identified by the feature detection methods: ME, Harris, BRISK, ORB, SURF, and KAZE.

Table S4. Area of a region between Ripley's K function and CSR.

Table S5. Area of a region between Ripley's K function and CSR (Augmented).
